# Implementing a package of noncommunicable disease interventions in the Republic of Moldova: two-year follow-up data

**DOI:** 10.1017/S1463423620000420

**Published:** 2020-09-30

**Authors:** Dylan Collins, Laura Inglin, Tiina Laatikainen, Angela Ciobanu, Ghenadie Curocichin, Virginia Salaru, Tatiana Zatic, Angela Anisei, Diana Chiosa, Maria Munteanu, Zinaida Alexa, Jill Farrington

**Affiliations:** 1 University of British Columbia, Vancouver, Canada; 2 Institute of Public Health and Clinical Nutrition, University of Eastern Finland, Kuopio, Finland; 3 National Institute for Health and Welfare, Helsinki, Finland; 4 Joint municipal authority for North Karelia health and social services (Siun sote), Joensuu, Finland; 5 World Health Organization Regional Office for Europe, Copenhagen, Denmark; 6 Family Medicine Department, Nicolae Testemitanu State Medical and Pharmaceutical University, Chisinau, Republic of Moldova; 7 Primary, Emergency and Community Health Policies Department, Ministry of Health, Labour and Social Protection, Chisinau, Republic of Moldova; 8 Department on Quality Management of Health Services, National Public Health Agency, Chisinau, Republic of Moldova; 9 Endocrinology Department, Nicolae Testemitanu State Medical and Pharmaceutical University, Chisinau, Republic of Moldova

## Abstract

Noncommunicable diseases (NCDs) are a growing challenge in the Republic of Moldova. A previously reported pilot cluster randomized controlled trial aimed to determine the feasibility of implementing and evaluating essential interventions for NCDs (e.g. cardiovascular risk scoring, hypertension management, statin treatment, etc.) in primary health care in the Republic of Moldova, with a view toward national scale up. One-year follow-up data (previously published) demonstrated modest improvements in NCD risk factor identification and management could be achieved. Herein, we report the second-year follow-up data and conclude that sustainable improvements in NCD risk factor control (e.g. hypertension control) can be achieved in primary health care in low resource settings by adapting existing resources (e.g. WHO PEN) and conducting focused clinical training and support. If scaled to a national level, these improvements in risk factor control could significantly translate to reductions in premature mortality from NCDs.

## Introduction

Noncommunicable diseases (NCDs) are a growing challenge in the Republic of Moldova, and their human, social, and economic impacts are immense. Similar to other low- and middle-income countries, the rates of premature death due to NCDs continue to be high with significant gender bias toward men relative to women (34% and 17%, respectively). (WHO, 2018) This is in part due to unfavorable population risk factors including high rates of smoking and alcohol use particularly amongst men, which are among the highest in the WHO European Region, poorly controlled hypertension, and gaps in primary health care. (NCD Risk Factor Collaboration, 2017)

Individual-level ‘best buy’ primary health care interventions, such as cardiovascular risk stratification, lipid-lowering treatment, blood pressure treatment, and healthy lifestyle counseling, are highly cost effective and reduce both premature mortality and economic burden. The per-person annual cost for NCD ‘best buy’ interventions ranges from 1 to 3 USD in low- and middle-income countries (LMIC). (Word Economic Forum, 2011) It is estimated that the use of these interventions in LMIC to reduce the mortality rate of ischemic heart disease and stroke by 10%, for example, would yield a savings of 25 billion USD across LMIC per year. (World Economic Forum, 2011)

For these reasons, among others, in their 2016–2018 Action Program, the Government of the Republic of Moldova made a clear commitment to strengthen primary health care and the implementation of best buy interventions, with a focus on cardiovascular diseases (CVD). As part of this mandate, a study was launched that aimed to determine the feasibility of implementing and evaluating essential interventions (e.g. cardiovascular risk scoring, hypertension management, statin treatment, etc.) in primary health care, with a view toward national scale up. This involved developing simplified clinical protocols from WHO Package of Essential NCD Interventions (WHO PEN) for the detection, prevention, and management of NCDs in primary care, in-person training of doctors and nurses, and technical and follow-up support. (Collins *et al.*, 2019) Ultimately, 20 primary health care facilities were recruited from across the country and 10 were randomized to the complex intervention (training, support, and simplified clinical guidance/protocols). The full study design and first year results have been published elsewhere. (Collins *et al.*, 2019; Laatikainen *et al.*, 2020)

In short, after one-year of follow-up, we concluded that WHO PEN protocols 1 and 2 were implementable in primary health care and that routine paper-based clinical data could be used to assess the effectiveness of these interventions. (Laatikainen *et al.*, 2020) Secondary to this, the study also evaluated the baseline performance of primary health care services and the change after one year. These analyses demonstrated that modest improvements in risk factor identification and management could be achieved in a relatively short period of time. (Laatikainen *et al.*, 2020)

While our original protocol for the evaluation of the pilot implementation detailed assessment of outcomes at one-year follow-up, in preparation for potential national scale up of the interventions, there was interest by key stakeholders in the longer-term impact and sustainability of the interventions (some of which ceased after 12 months). Not least because the prevention and management of NCDs require long term control of risk factors. Thus, the *a priori* outcomes were assessed again at second year using the previously described methodology. (Collins *et al.*, 2019; Laatikainen *et al.*, 2020) Herein, we report the second-year follow-up data from the aforementioned feasibility study.

## Methods

**Figure 1.**
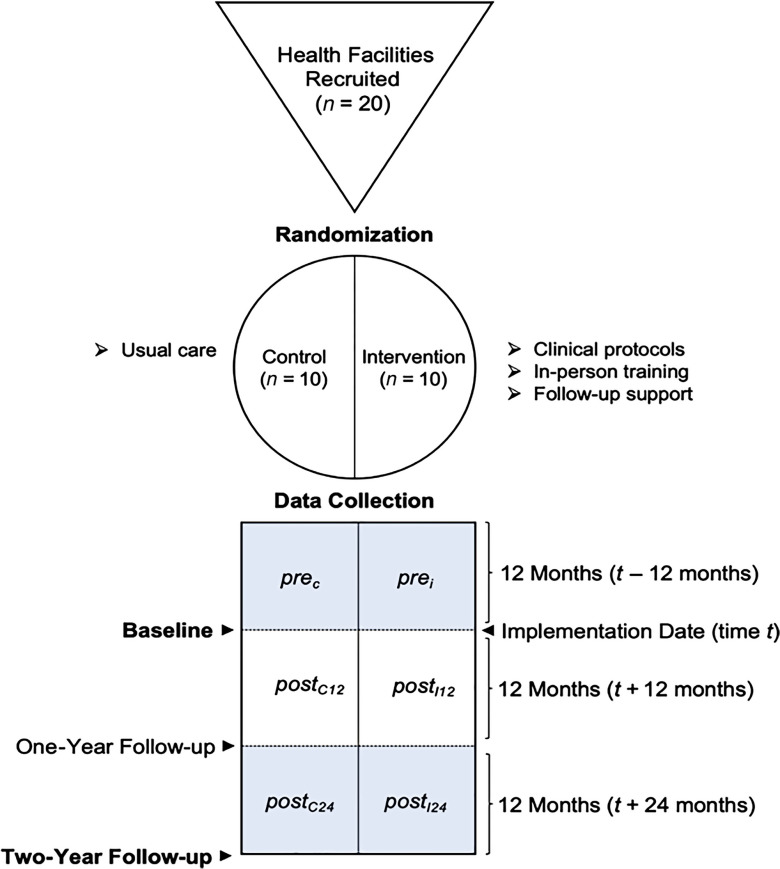
Overview of study design illustrating the baseline, one year, and two-year follow-up points

We previously published our study protocol which describes the methodology in detail. (Collins *et al.*, 2019) The one-year follow-up data are published in the European Journal of Public Health, along with additional methodological information. (Laatikainen *et al.*, 2020) In short, we conducted a feasibility study of a cluster randomized controlled trial of primary health care facilities (*n* = 20) which were randomly allocated to intervention (*n* = 10) or control (*n* = 10) (Figure 1). The intervention is described in detail elsewhere, but in brief consisted of adapted clinical protocols, training of primary care clinicians, technical and follow-up support. The technical and follow-up support lasted for 12 months after training after which no further support was provided to intervention clinics. Herein, we report the same indicators reported in the first-year follow-up, using data collected at 24 months after the intervention began. Data were manually extracted from randomly selected paper-based clinical records into a standardized form and indicators were calculated from these data (Table 1).

**Table 1.** Indicator and risk factor definitions

Term	Definition
Process Indicators	
HbA1c measure for DM patients	Proportion of diabetic* patients with at least one documented HbA1c measurement during the last 12 months
Smoking status recorded	Proportion of patients with smoking status recorded
BP measured regularly	Proportion of patients with two documented blood pressure measurements during the last 12 months
ESC SCORE documented	Proportion of patients aged 40 years or more with a documented total cardiovascular risk score
Statin prescribed for CVD patients	Proportion of patients with existing cardiovascular disease* prescribed a statin
Statin prescribed for DM patients	Proportion of diabetic* patients aged over 40 prescribed a statin
Aspirin prescribed for CVD patients	Proportion of patients with existing cardiovascular disease* prescribed aspirin
Outcome Indicators	
BP at normal range	Proportion of patients whose last two recorded BP measurements were at normal range (SBP < 140 mmHg and DBP < 90 mmHg), of which at least the most recent one during the last 12 months
Hypertensive patients with BP control	Proportion of hypertensive* patients whose last two recorded blood pressure measurements were at normal range (SBP < 140 mmHg and DBP < 90 mmHg), of which at least the most recent measurement was during the last 12 months
Patients with elevated BP	Proportion of patients whose last two SBP readings were ≥ 140 mmHg or DBP ≥ 90 mmHg, of which at least the most recent measurement was during the last 12 months

Abbreviations: ESC SCORE = the Systematic Coronary Risk Evaluation score of the European Society of Cardiology; SBP = systolic blood pressure; DBP = diastolic blood pressure; HbA1c = glycosylated hemoglobin; DM = diabetes mellitus; BP = blood pressure; CVD = cardiovascular disease

* Diagnoses written either using ICD-10 codes or as names of diseases in the section of permanent diagnoses in the patient records are taken into account

Separate logistic regression models were used for intervention and control clinics to determine changes in dichotomous outcomes between baseline and follow-up and to assess interactions between allocation group and time. Multivariate linear regression models were used to evaluate continuous variables and to assess interaction effects. All results were adjusted for age and sex. *P*-values of <0.05 were considered statistically significant.

## Results

At two-year follow-up, a total of 2598 patients records were included; 1329 from the intervention clinics and 1269 from the control clinics. Gender and age distributions were similar between the intervention and control clinics (Table 2). A small majority of patients in both allocation groups were female: 58.9% and 57.5% in intervention and control clinics, respectively (Table 2). The median age of records sampled were similar between intervention and control clinics: 60 (IQR 49–67) and 59 (IQR 46–66) years, respectively. For both allocation groups, the median age was slightly higher at two-year follow-up than at baseline (57 and 56, respectively) (Table 2). We also include the one-year follow-up data for context (Tables 2 and 3), although previously reported.

**Table 2.** Gender and age distribution of patient records sampled at baseline, 1 year, and 2 years follow-up

	Intervention			Control		
	Baseline *n* = 1174	One-year Follow-up *n* = 1329	Two-year Follow-up *n* = 1329	Baseline *n* = 995	One-year Follow-up *n* = 1256	Two-year Follow-up *n* = 1269
Gender						
Female, % (n)	62.4 (733)	60.6 (805)	58.9 (783)	61.8 (615)	60.6 (761)	57.5 (730)
Male, % (n)	37.6 (441)	39.4 (524)	41.1 (546)	38.2 (380)	39.4 (495)	42.5 (539)
Age, Median (IQR)	57 (44-65)	59 (46-67)	60 (49-67)	56 (42–64)	58 (43–65)	59 (46-66)
18–39 years, % (n)	20.2 (237)	18.0 (239)	14.4 (191)	21.7 (216)	20.2 (254)	17.3 (220)
40–49 years, % (n)	11.8 (138)	11.7 (156)	11.6 (154)	12.8 (127)	12.3 (155)	12.9 (164)
50–59 years, % (n)	26.7 (313)	21.0 (279)	20.5 (273)	25.1 (250)	22.7 (285)	20.7 (263)
60–69 years, % (n)	28.5 (335)	34.4 (457)	36.1 (480)	29.9 (298)	32.1 (403)	32.5 (413)
70+ years, % (n)	12.9 (151)	14.9 (198)	17.4 (231)	10.5 (104)	12.7 (159)	16.5 (209)

Abbreviations: IQR, interquartile range

**Table 3.** Outcome indicators at baseline, one-year follow-up, and two-year follow-up in intervention and control clinics

Characteristic	Baseline	First Follow-up	Second Follow-up	Baseline versus First Follow-up	First Follow-up versus Second Follow-up	Baseline vs 2nd Follow-up
	% (*n*/N)	% (*n*/N)	% (*n*/N)	OR (95 % CI)*	OR (95 % CI)*	OR (95 % CI)*
Intervention						
Process indicators						
HbA1c measured from DM patients	33.5 (58/173)	63.3 (169/267)	75.3 (174/231)	3.444 (2.301–5.154)***	1.809 (1.224–2.676)**	6.034 (3.896–9.345)***
Smoking status recorded	50.6 (594/1174)	66.0 (877/1329)	67.2 (893/1329)	1.879 (1.598–2.209)***	***1.053 (0.894–1.240)***	1.964 (1.667–2.313)***
BP measured regularly	63.5 (746/1174)	64.9 (863/1329)	70.4 (935/1329)	***0.963 (0.804–1.154)***	1.249 (1.045–1.493)**	***1.198 (0.997–1.429)***
ESC Score documented	55.9 (524/937)	36.5 (398/1090)	55.4 (630/1138)	0.453 (0.376–0.546)***	2.248 (1.889–2.675)***	***1.056 (0.881–1.266)***
High risk patients (ESC Score≥10%)	9.0 (47/524)	12.8 (51/398)	22.5 (142/630)	1.194 (0.763–1.869)	1.792 (1.241–2.588)**	2.163 (1.489–3.142)***
Statin prescribed for CVD patients	15.9 (64/402)	19.2 (89/464)	27.7 (156/563)	***1.258 (0.883–1.791)***	1.611 (1.198–2.166)**	2.071 (1.494–2.872)***
Statin prescribed for high-risk DM patients	17.9 (30/168)	19.4 (51/263)	28.1 (64/228)	***1.111 (0.674–1.832)***	1.614 (1.059–2.461)**	1.870 (1.141–3.064)**
Aspirin prescribed for CVD patients	74.1 (298/402)	70.0 (325/464)	75.8 (427/563)	0.789 (0.582–1.070)	1.278 (0.964–1.695)	***1.011 (0.747–1.368)***
Outcome indicators						
BP at normal range	42.1 (429/1019)	67.4 (695/1031)	77.5 (907/1170)	3.577 (2.943–4.348)***	1.705 (1.408–2.065)***	6.710 (5.453–8.256)***
HT patients with BP control	21.7 (134/617)	61.9 (458/740)	73.1 (572/782)	5.846 (4.588–7.449)***	1.666 (1.340–2.070)***	9.943 (7.742–12.770)***
Patients with elevated BP	33.5 (323/964)	12.6 (126/1003)	6.9 (78/1132)	0.249 (0.196–0.316)***	0.508 (0.377–0.684)***	0.120 (0.091–0.159)***
Control						
Process indicators						
HbA1c measured from DM patients	16.3 (20/123)	49.0 (94/192)	72.9 (161/221)	5.074 (2.897–8.888)***	2.811 (1.863–4.239)***	14.536 (8.196–25.782)***
Smoking status recorded	53.0 (527/995)	62.5 (785/1256)	55.6 (705/1269)	1.461 (1.231–1.734)***	0.735 (0.626–0.863)***	***1.062 (0.896–1.258)***
BP measured regularly	52.3 (520/995)	58.6 (736/1256)	66.9 (849/1269)	1.251 (1.042–1.502)**	1.429 (1.201–1.702)***	1.790 (1.485–2.158)***
ESC Score documented	62.6 (488/779)	46.2 (463/1002)	42.2 (443/1049)	0.512 (0.420–0.624)***	***0.871 (0.730–1.040)***	0.449 (0.369–0.546)***
High risk patients (ESC Score≥10%)	8.2 (40/488)	9.1 (42/463)	10.4 (46/443)	0.958 (0.585–1.570)	0.974 (0.610–1.558)	***1.021 (0.635–1.641)***
Statin prescribed for CVD patients	20.1 (69/344)	13.9 (67/483)	12.5 (63/504)	0.639 (0.441–0.924)**	***0.886 (0.612–1.283)***	0.568 (0.391–0.825)**
Statin prescribed for high-risk DM patients	27.5 (33/120)	13.4 (25/187)	12.3 (27/219)	0.411 (0.229–0.735)**	0.884 (0.492–1.589)	0.361 (0.203–0.641)**
Aspirin prescribed for CVD patients	79.7 (274/344)	70.4 (340/483)	72.0 (363/504)	0.612 (0.441–0.850)**	1.071 (0.811–1.414)	0.657 (0.473–0.912)**
Outcome indicators						
BP at normal range	53.2 (439/825)	61.2 (585/956)	72.2 (775/1074)	1.521 (1.246–1.855)***	1.721 (1.422–2.082)***	2.764 (2.253–3.392)***
HT patients with BP control	28.5 (134/470)	51.5 (336/652)	65.9 (482/731)	2.661 (2.067–3.426)***	1.841 (1.473–2.274)***	4.940 (3.834–6.365)***
Patients with elevated BP	25.0 (188/752)	15.7 (142/902)	12.1 (126/1045)	0.523 (0.408–0.672)***	0.722 (0.556–0.938)**	0.368 (0.284–0.476)***

* Age and gender adjusted; ** *p* < 0.05; *** *p* < 0.001; ***p for interaction < 0.05***

**Figure 2.**
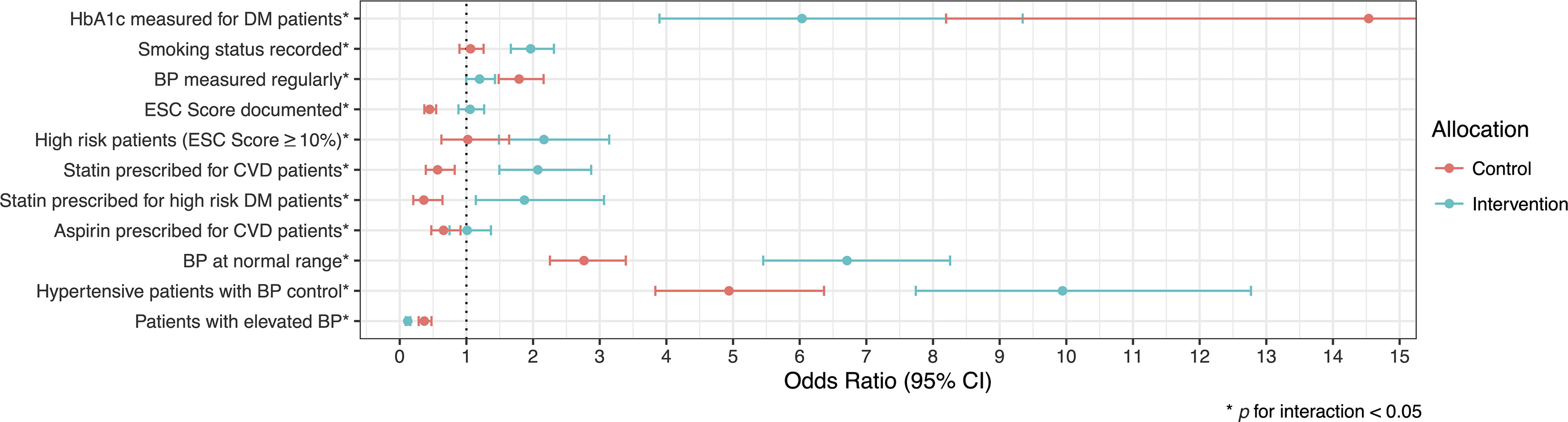
Odds of achieving outcomes (odds ratio with 95% CI) at two-year follow-up compared to baseline for both intervention and control clinics

All indicators measured were significantly different (*p* for interaction < 0.05) between intervention and control clinics at 24 months, although not all improved in the intervention clinics (Table 3). The intervention clinic indicators that were significantly improved relative control clinics at 24 months were: recording of smoking status (OR 1.96 95% CI 1.67, 2.31), documentation of high risk patients (OR 2.16 95% CI 1.49, 3.14), statin prescribing for CVD patients (OR 2.07 95% CI 1.49, 2.87), statin prescribing for high risk diabetes (DM) patients (those age 40 years or older) (OR 1.87 95% CI 1.14, 3.06), prevalence of normal blood pressure (OR 6.71 95% CI 5.45, 8.26), prevalence of elevated blood pressure (OR 0.12 95% CI 0.09, 0.16), and blood pressure control amongst patients with diagnosed hypertension (OR 9.94 95% CI 7.74, 12.77) (Table 3, Figure 2).

By 24 months, both intervention and control clinics improved their measurement of (hemoglobin A1c) HbA1c amongst patients with DM, but the control clinics significantly improved more than the intervention clinics: OR 14.54 (95% CI 8.20, 25.78) and 6.03 (95% CI 3.90, 9.35), respectively.

Whereas the intervention clinics had no change in regular blood pressure measurement over two years, the control clinics significantly improved to similar rates of that seen in the intervention clinics: OR of 1.20 (95% CI 1.00, 1.43) and 1.79 (95% CI 1.49, 2.16), respectively. This equates to 70.4% and 66.9% of intervention and control clinic patients with regular blood pressure measurement, respectively (Table 3).

There was no improvement in documentation of ESC Score in intervention clinics, and control clinics significantly worsened: OR 1.06 (95% CI 0.88, 1.27) and 0.45 (95% CI 0.37, 0.55), respectively (Table 3). This was also true for aspirin prescribing among patients with existing CVD (Table 3).

## Discussion

After two years of implementation, significant improvements were seen in both the prevalence of normal blood pressure and the proportion of patients with hypertension who achieved blood pressure targets as a result of focused training, simplified clinical protocols, and implementation support. The odds that a patient with hypertension had blood pressure controlled was 10 times higher two years after implementation than at baseline. While the odds also increased in control clinics, the odds ratio in the intervention clinics was double that of control clinics (OR 10 and 5, respectively). Furthermore, in intervention clinics, the process indicators either significantly improved (recording of smoking status, prescribing statins for patients with existing CVD or high risk DM patients) or remained the same (regular blood pressure measurement, documentation of ESC risk score, aspirin prescribing for CVD patients). This is in the context of four of the control clinic indicators worsening over the two-year timeframe (documentation of ESC risk score, statin prescribing in both CVD patients and high-risk DM patients, and aspirin prescribing for CVD patients). Both HbA1c measurement in DM patients and regular measurement of blood pressure failed to improve to the same degree as seen in the control clinics. Of those with a documented risk score, the odds of being high risk (ESC Score ≥10%) were significant greater in the intervention clinics compared to the control clinics. This may be due to increased targeting and follow-up of high risk patients, or a selection bias in who clinicians decide to calculate a risk score.

Significant improvement was seen for several indicators in control clinics (HbA1c measurement in patients with DM, regular measurement of blood pressure, the prevalence of normal blood pressure, and blood pressure control amongst hypertensive patients). This demonstrates that the quality of usual care improved during the two-year study timeframe. We are aware of several national achievements during the study timeframe that may partially explain the improvement in usual care: Ministry of Health approval of WHO PEN protocols for use in all primary health care clinics, establishment of a continuing education module for family physicians based on WHO PEN protocols, additional training projects aimed at NCD control in primary health care, public health awareness and self-care campaigns for patients with NCDs, and the widespread provision of self-auditing tools to primary health care for assessment of NCD care. Nonetheless, due to the randomized controlled design, we can be confident in the attribution of intervention effects to the intervention alone despite improvements in control clinics.

Overall, these findings are consistent with existing literature that demonstrate the positive impact of continuing medical education (CME) on both clinician behavior and patient outcomes. In particular our focus on interactive learning methods, practical simulation of skills, clinic-level contextualization, and ongoing/follow-up technical support are known to increase the overall effect of CME on patient outcomes. (Cervero and Gaines, 2015) Furthermore, the impact on patient outcomes are both statistically, but more importantly, clinically significant. For example, target 6 of the global voluntary targets to reduce premature deaths from NCDs by 25% by 2025 is a 25% relative reduction in prevalence of raised blood pressure. (WHO, 2020) While this was achieved in both intervention and control clinics, the intervention clinics had a significantly greater relative reduction in raised blood pressure compared to control clinics (79% and 52% relative reduction, respectively). Scaling the use of evidence-based CME, clinical decision support tools, and ongoing support in primary health care could therefore have a significant impact on reducing premature mortality from NCDs in the Republic of Moldova. (Blood Pressure Lowering Treatment Trialists’ Collaboration, 2014)

The strengths of this work include an a priori design, a scalable methodological approach, and representative selection of primary health care clinics from across different regions of the country. These results provide evidence from a real-world health system for the longer-term impacts on important patient outcomes, such as blood pressure control amongst patients with hypertension, and these are sustainable beyond the end of the implementation support (12 months). This evidence can therefore inform the national policies of the Republic of Moldova, as well as provide a methodological example to other countries. Our work is limited in that we did not include patient reported outcomes, quality of life measurements, or morbidity and mortality data. Although we did not assess the cost effectiveness of the intervention in the Republic of Moldova, WHO PEN itself is based on cost effective interventions and well established ‘best-buy’ interventions. (WHO, 2017; WHO, 2010) As with any study, our indicators are limited by the quality of the data, which can be variable when extracting from written paper-based clinical records. In future, a step wedge design may be beneficial with respect to national scale up as well as determining sample sizes that ensure adequate power to analyze subpopulations such as patients with diabetes. (Hemming *et al.*, 2015)

## Conclusion

Sustainable improvements in NCD risk factor control can be achieved in primary health care in low resource settings by adapting existing resources (e.g. WHO PEN) and conducting focused clinical training and support within a two-year time period. If scaled to a national level these could significantly translate to reductions in premature mortality from NCDs.
